# Prognosis and Toxicity Stratified by Best Tumor Burden Change in Japanese Patients With Advanced Melanoma Treated With First‐Line Programmed Cell Death Protein 1 Monotherapy

**DOI:** 10.1111/1346-8138.17938

**Published:** 2025-09-05

**Authors:** Ken Horisaki, Shusuke Yoshikawa, Wataru Omata, Arata Tsutsumida, Yoshio Kiyohara

**Affiliations:** ^1^ Department of Dermatology Shizuoka Cancer Center Shizuoka Japan; ^2^ Department of Dermatology Nagoya University Graduate School of Medicine Nagoya Japan

**Keywords:** immune‐check point inhibitor, melanoma, nivolumab, pembrolizumab

## Abstract

Immune checkpoint inhibitors (ICIs) have significantly improved outcomes in patients with advanced malignant melanoma (MM). However, more than half of patients receiving anti‐programmed cell death protein‐1 (PD‐1) antibody monotherapy still fail to respond, with response rates varying by race and melanoma subtype. Additionally, immune‐related adverse events (irAEs) remain a major concern. Although the best overall response based on Response Evaluation Criteria in Solid Tumors version 1.1 is commonly used in clinical trials to assess efficacy, its utility in predicting prognosis and toxicity in real‐world clinical settings remains unclear. This retrospective cohort study conducted at Shizuoka Cancer Center in Japan evaluated the association between best tumor burden change (BTBC) in target lesions and prognosis or toxicity among Japanese patients with stage IV MM who received PD‐1 monotherapy as first‐line treatment. A total of 115 patients were analyzed. Prognosis improved proportionally with reductions in tumor burden from baseline. No significant difference was observed in overall survival between the partial response group and the stable disease group (*p* = 0.833). However, BTBC < 0% was a significant indicator of a favorable long‐term prognosis (*p* < 0.001). The development of new lesions indicated poor prognosis; however, BTBC ≥ 0% represented poor prognosis regardless of new lesions. Regarding toxicity, the incidence of any‐grade irAEs was significantly higher in the BTBC < 0% group than in the BTBC ≥ 0% group (*p* < 0.001), suggesting that greater tumor shrinkage correlated with increased toxicity. These findings indicate that BTBC may serve as a more accurate predictor of prognosis and toxicity than best overall response in clinical practice. Incorporating BTBC into treatment planning and toxicity monitoring could improve management of stage IV MM, though future prospective studies are needed to establish standardized BTBC criteria.

## 1 Introduction

Immune checkpoint inhibitors (ICIs) serve as a cornerstone of systemic therapy for advanced malignant melanoma (MM), alongside molecularly targeted drugs, and have significantly improved patient prognosis. Current ICI regimens for advanced MM include monotherapy with anti‐programmed cell death protein‐1 (PD‐1) antibodies such as nivolumab or pembrolizumab, as well as combination therapy with nivolumab and ipilimumab. Despite these advances, several clinical trials [1, 2, 3] have shown that more than half of patients with MM do not respond to ICIs, with response rates differing by race and melanoma subtype. For example, trials predominantly conducted in Western populations have reported response rates of approximately 40%–50% for PD‐1 monotherapy in stage IV MM [1, 2, 4]. In contrast, Japanese patients with MM exhibit lower response rates, ranging from approximately 15%–30% [5, 6]. Toxicity also plays a critical role in ICI selection. Immune‐related adverse events (irAEs) occur in approximately 60%–80% of patients with MM receiving PD‐1 monotherapy [1, 7, 8], and serious irAEs affect approximately 10%–20% of these patients [1, 2, 7, 8, 9]. These adverse events significantly influence therapeutic decision‐making.

Although clinical trials assess short‐term treatment efficacy using standardized criteria such as Response Evaluation Criteria in Solid Tumors (RECIST) version 1.1 [10], immune‐RECIST [11], and immune‐related RECIST [12], real‐world clinical practice often requires a different approach [13]. In practice, decisions to continue treatment depend on a complex assessment that integrates both objective therapeutic outcomes and patient‐specific subjective factors. Among objective indicators, metrics that can predict prognosis and toxicity are particularly valuable for guiding treatment continuation. However, the utility of best overall response as defined by RECIST criteria in predicting long‐term outcomes and toxicity in clinical settings remains uncertain. This study aimed to evaluate the association between best tumor burden change (BTBC) in target lesions and both prognosis and toxicity in Japanese patients with stage IV MM receiving PD‐1 monotherapy as first‐line treatment. The study also sought to explore more practical, objective criteria to guide clinical decision‐making regarding determining PD‐1 monotherapy efficacy.

## 2 Methods

### 2.1 Study Population and Data Collection

This retrospective cohort study was conducted at Shizuoka Cancer Center, Japan. Medical records were reviewed for patients with stage IV MM who received PD‐1 monotherapy as first‐line therapy between February 2012 and December 2024. The inclusion criteria were as follows: (i) pathologically confirmed MM; (ii) primary sites including all skin, mucosa, uvea, or unknown primary sites; (iii) stage IV classification according to the 8th edition of the American Joint Committee on Cancer (AJCC) staging system [14]; (iv) administration of either nivolumab or pembrolizumab monotherapy as first‐line systemic therapy; and (v) completion of at least one imaging test (computed tomography or magnetic resonance imaging) to assess the efficacy of PD‐1 monotherapy. For AJCC staging, the cutaneous MM criteria were applied for unclassifiable genital, anal, or urinary tract tumors. Patients without target lesions before PD‐1 monotherapy were excluded.

The collected clinical data included participants' age, sex, Eastern Cooperative Oncology Group performance status (ECOG‐PS), primary tumor location, history of surgical resection of the primary tumor and use of adjuvant therapy, stage IV classification, B‐rapidly accelerated fibrosarcoma (BRAF) mutation status, presence of irAEs, and lactate dehydrogenase (LDH) levels at PD‐1 initiation.

### 2.2 Definition of BTBC in Target Lesions

BTBC was determined according to the methodology described in the RECIST version 1.1 [10]. Up to five measurable target lesions, with a maximum of two lesions per organ, were selected. When selecting target lesions from multiple candidates, larger lesions were prioritized in descending order of size. This approach was adopted to minimize the impact of potential measurement errors. The sum of diameters for all selected lesions was calculated—using the longest diameter for solid tumors and the short axis for lymph nodes. Solid lesions required a minimum diameter of 10 mm, whereas lymph nodes had to be at least 15 mm in short‐axis diameter to qualify as target lesions. The baseline tumor burden was assessed before the initiation of PD‐1 monotherapy using this procedure. After starting PD‐1 monotherapy, the diameters of target lesions were re‐measured every 2–12 weeks. The percentage change from baseline was calculated at each follow‐up. The largest observed reduction in tumor size during the course of treatment was recorded as the BTBC.

### 2.3 Efficacy Assessment

The primary outcome was overall survival (OS) for each BTBC group. The secondary outcome was the incidence of irAEs. A sub‐analysis was conducted to compare OS based on the presence or absence of new metastatic lesions. OS was defined as the time from initiation of PD‐1 monotherapy to death from any cause or final follow‐up. Treatment response was assessed using the RECIST version 1.1, with the best overall response for each treatment representing the most favorable assessment of treatment response. The objective response rate (ORR) was defined as the proportion of participants achieving complete response (CR) or partial response (PR). Finally, irAEs were graded according to the Common Terminology Criteria for Adverse Events (CTCAE) version 5.0 [15].

### 2.4 Statistical Analysis

To determine the optimal BTBC cutoff value for predicting prognosis, receiver operating characteristic (ROC) curves were constructed using the DeLong model. For this analysis, the endpoint was survival status on each day over a 5‐year period from the start of ICI treatment. A range of potential cutoff values was generated from these daily ROC curves. The Kaplan–Meier method was applied for each of these potential cutoff values, and the value that showed the most significant difference between the two survival curves, indicated by the smallest *p*‐value in the log‐rank test, was selected as the final cutoff value. This analysis was performed for both the entire patient cohort and a subgroup comprising patients categorized as having a PR or stable disease (SD) according to the RECIST v1.1. OS for each group, based on the optimal cutoff value, was estimated using the Kaplan–Meier method and compared using the log‐rank test. Patients who remained alive at the final follow‐up or were lost to follow‐up were censored. For a more detailed evaluation, patients were further divided into 30% BTBC intervals. This approach was adopted to better assess the prognostic significance of BTBC across a granular spectrum, particularly for comparing outcomes around the conventional RECIST threshold of −30%, which distinguishes PR from SD, and the new clinically relevant threshold of 0%. Median OS for each group was plotted using the Kaplan–Meier method, and the relationship between BTBC and median OS was calculated as an exponential function using the least squares method. Comparisons of the incidence of new lesions and irAEs between BTBC groups were performed using the chi‐squared test or Fisher's exact test. To clarify the relationship between BTBC and irAEs, a multivariate logistic regression analysis was conducted, including BTBC < 0% and treatment duration as independent variables and the occurrence of any‐grade irAEs as the dependent variable. Both univariate and multivariate analyses were performed to assess the independent contributions of each factor. The treatment duration was defined as the time from the first administration of the ICI to the first imaging evaluation following the final administration. Statistical significance was set at *p* < 0.05. All analyses were performed using EZR version 1.55 (Saitama Medical Center, Jichi Medical University, Saitama, Japan), a graphical interface for R software (The R Foundation for Statistical Computing, Vienna, Austria).

### 2.5 Ethics Statement

This retrospective cohort study was approved by the Institutional Review Board of Shizuoka Cancer Center (approval number: J2025‐73). The requirement for informed consent was waived due to the retrospective observational nature of the study. All personal data were handled in accordance with the ethical standards of the 1964 Declaration of Helsinki and its subsequent amendments.

## 3 Results

### 3.1 Characteristics of Patients With MM Treated With PD‐1 Monotherapy

A total of 115 patients underwent PD‐1 monotherapy as first‐line therapy, as summarized in Table 1. The overall median follow‐up period was 13.0 months. The most common primary site of MM was cutaneous, excluding acral, in 43 patients (37.4%), followed by mucosal in 42 (36.5%), and acral in 18 (15.7%). Approximately half of the patients had elevated LDH levels (*n* = 56, 48.7%), and more than half did not have BRAF gene mutations (*n* = 76, 66.1%). As a PD‐1 monotherapy, 87.8% of patients were treated with nivolumab and 12.2% with pembrolizumab.

**TABLE 1:** Baseline characteristics of patients with malignant melanoma.

Characteristic	Number of patients (%)
Patients *n* (%)	115 (100)
Age, median [range]	70.0 [27.0–92.0]
Sex	
Male	61 (53.0)
Female	54 (47.0)
ECOG‐PS	
0–1	102 (88.7)
≥ 2	13 (11.3)
Subtype	
Cutaneous	43 (37.4)
Acral	18 (15.7)
Mucosal	42 (36.5)
Uveal	3 (2.6)
Unknown	9 (7.8)
Details of stage IV	
M1a	25 (21.7)
M1b	20 (17.4)
M1c	64 (55.7)
M1d	6 (5.2)
LDH value	
< ULN	59 (51.3)
≥ ULN	56 (48.7)
BRAF	
Mutant	12 (10.4)
Wild	76 (66.1)
Not investigated	27 (23.5)
Number of organs involved	
1	61 (53.0)
2	26 (22.6)
≥ 3	28 (24.3)
Regimen of PD‐1monotherapy	
Nivolumab	101 (87.8)
Pembrolizumab	14 (12.2)
Surgery for primary site	
Yes	78 (67.8)
Adjuvant therapy	
Yes	23 (20.0)
Outcome	
Alive	30 (26.1)
Dead	85 (73.9)

Abbreviations: BRAF, B‐rapidly accelerated fibrosarcoma; ECOG‐PS, Eastern Cooperative Oncology Group Performance Status; LDH, lactate dehydrogenase; PD‐1, anti‐PD‐1 antibody; ULN, upper limit of normal.

### 3.2 BTBC in Target Lesions and Objective Response

**FIGURE 1:**
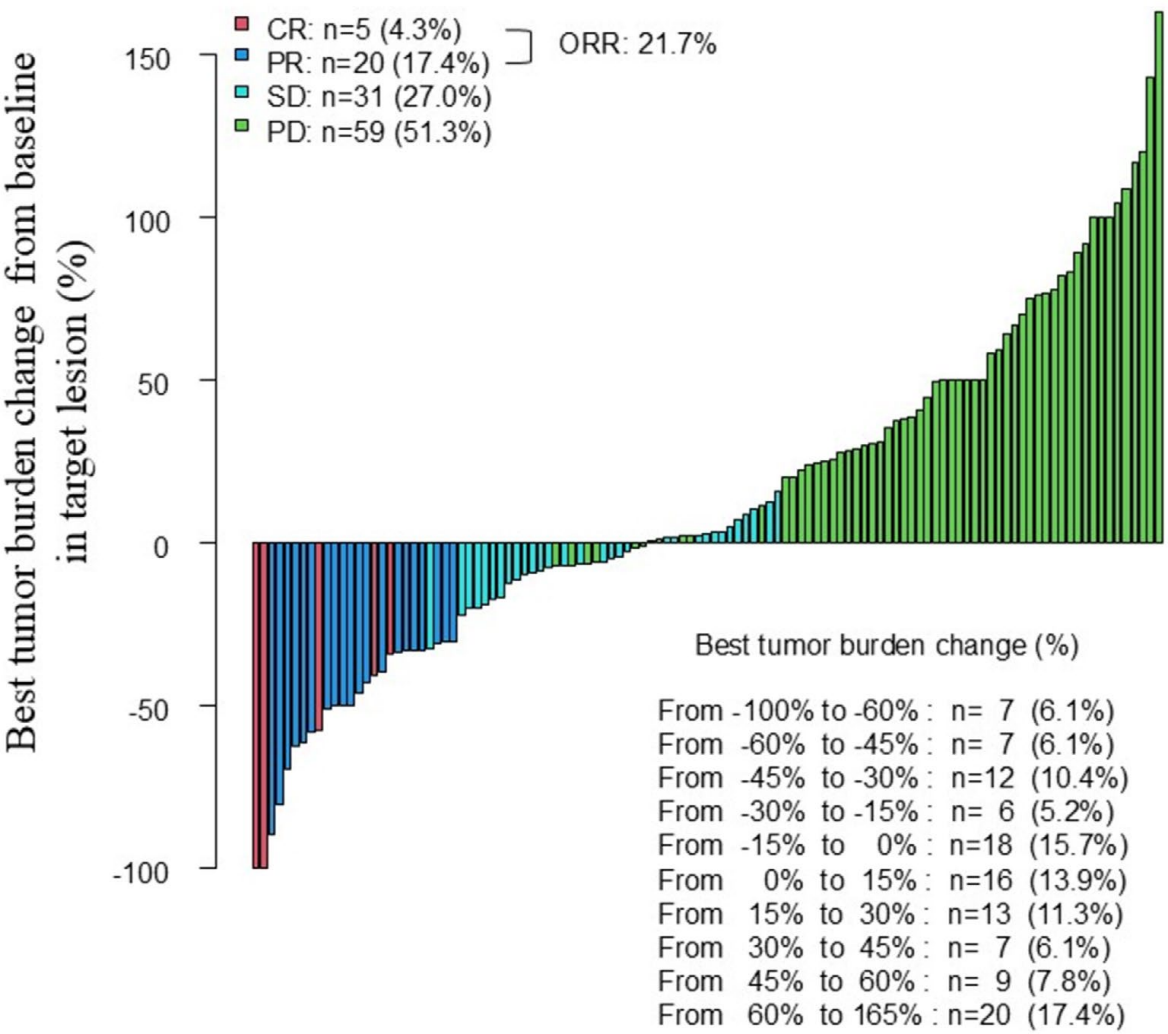
Waterfall plot of best tumor burden change (BTBC) in the target lesion and best overall response in 115 patients with melanoma treated with anti‐PD‐1 antibody monotherapy. The BTBC ranged from −100% to 165%. Red indicates complete response (CR), blue indicates partial response (PR), light blue indicates stable disease (SD), and green indicates progressive disease. The best overall response was CR in five patients (4.3%), PR in 20 (17.4%), stable disease (SD) in 31 (27.0%), and progressive disease in 59 (51.3%). The overall objective response rate was 21.7%.

The BTBC from baseline is summarized in Figure 1, with values ranging from −100% to 165%. Fifty patients (43.5%) had a decrease in BTBC (BTBC < 0% group), and 65 patients (56.5%) had an increase in BTBC (BTBC ≥ 0% group). The best overall responses were as follows: CR in five patients (4.3%), PR in 20 (17.4%), SD in 31 (27.0%), and progressive disease in 59 (51.3%). The overall objective response rate was 21.7%.

### 3.3 Overall Survival

**FIGURE 2:**
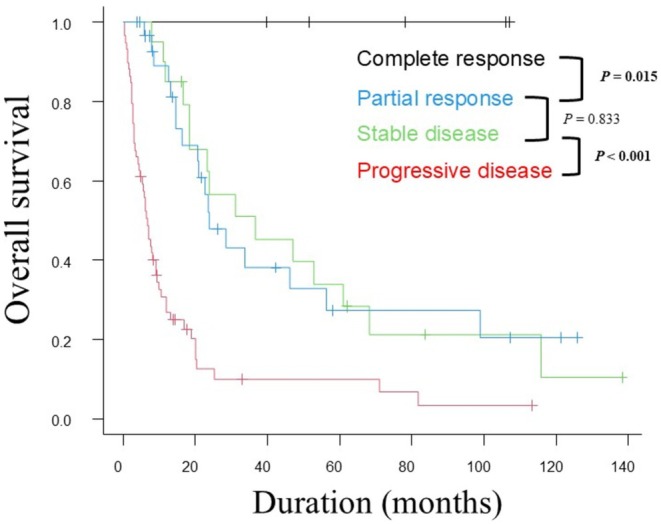
Kaplan–Meier analysis of overall survival (OS) by best overall response. The median OS by best overall response was not reached in the complete response (CR) group, was 36 months (95% confidential interval [CI]: 18.3–61.0) in the partial response (PR) group, 23 months (95% CI: 16.2–56.4) in the stable disease (SD) group, and 6.7 months (95% CI: 4.4–9.1) in the progressive disease group (*p* < 0.001). The CR group had significantly better OS than the PR group (*p* = 0.015), and the SD group had significantly better OS than the progressive disease group (*p* < 0.001). However, no significant difference in OS was observed between the PR and SD groups (*p* = 0.833).

**FIGURE 3:**
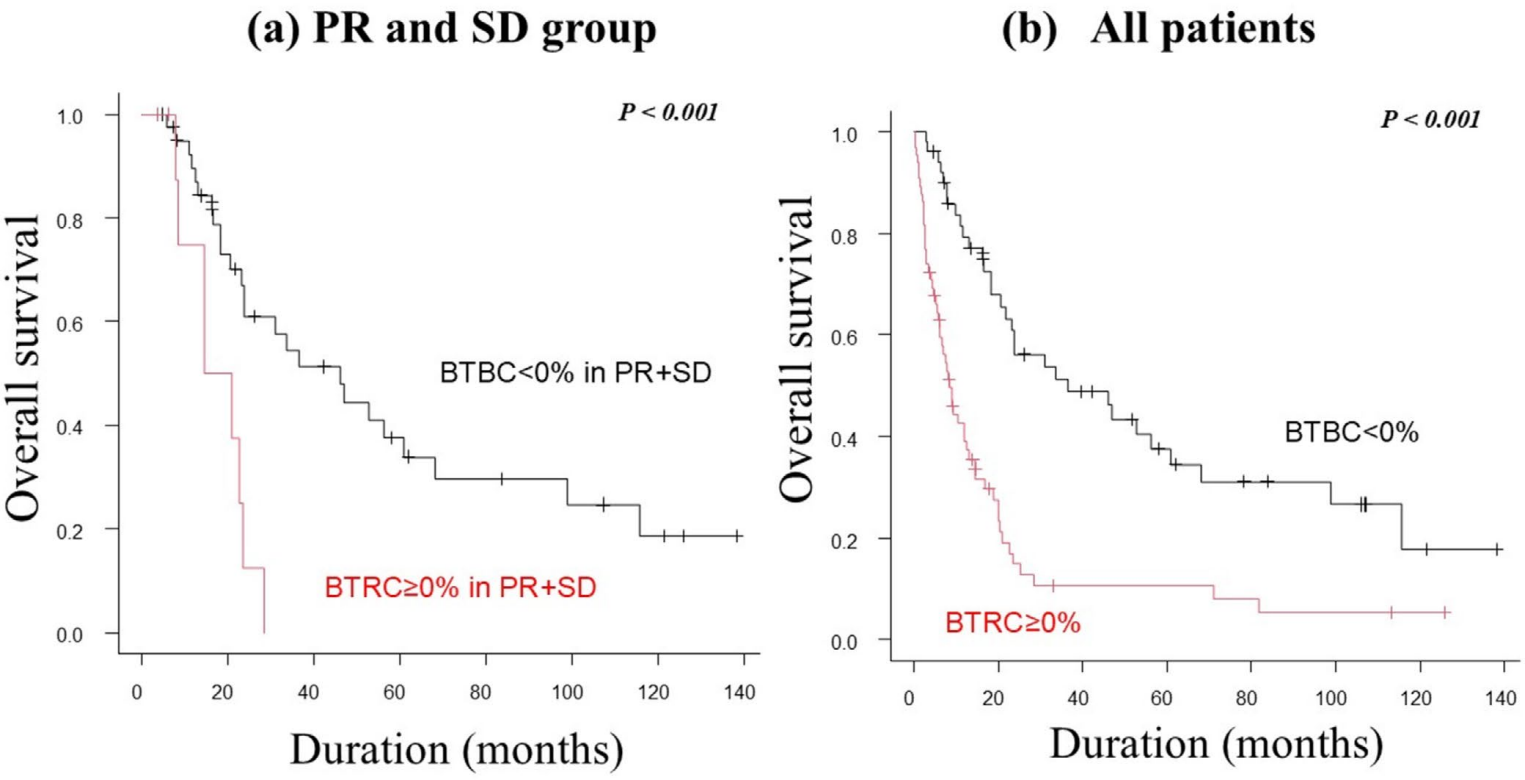
Kaplan–Meier analysis of overall survival (OS) between the best tumor burden change (BTBC) ≥ 0% and BTBC < 0% groups. (a) Kaplan–Meier analysis of OS in the BTBC ≥ 0% and BTBC < 0% groups, with best overall response determined to be partial response (PR) or stable disease (SD). The BTBC < 0 group had a significantly better OS than the BTBC ≥ 0 (median OS: 36.4 months vs. 14.6 months, *p* < 0.001). (b) Kaplan–Meier analysis of OS in the BTBC ≥ 0% and BTBC < 0% group in all patients. The BTBC < 0% group also had significantly better OS than the BTBC ≥ 0% group (median OS: 36.5 months vs. 8.5 months, *p* < 0.001).

The median OS by best overall response was not reached in the CR group, was 36 months (95% confidential interval [CI]: 18.3–61.0) in the PR group, 23 months (95% CI: 16.2–56.4) in the SD group, and 6.7 months (95% CI: 4.4–9.1) in the progressive disease group (*p* < 0.001) (Figure 2). The CR group had significantly better OS than the PR group (*p* = 0.015), and the SD group had significantly better OS than the progressive disease group (*p* < 0.001). However, no significant difference in OS was observed between the PR and SD groups (*p* = 0.833). The ROC curve for OS in the combined PR and SD group (PR + SD group) indicated an optimal cutoff value of −2.8%, with an area under the curve (AUC) of 0.649, sensitivity of 0.326, and specificity of 0.993 (Figure S1). For all patients, the ROC curve showed that the optimal cutoff value for OS was −2.9%, with an AUC of 0.803, sensitivity of 0.776, and specificity of 0.766 (Figure S2). For simplicity, the cutoff was set at 0%, and the OS was compared between the BTBC < 0% (*n* = 41) and BTBC ≥ 0% (*n* = 10) subgroups within the PR + SD group (Figure 3a). The sensitivity and specificity of a BTBC cutoff of 0% were 0.375 and 0.923, respectively, in PR + SD patients, and 0.712 and 0.690, respectively, in all patients (Figures S1 and S2). The BTBC < 0% subgroup had significantly better OS than the BTBC ≥ 0% subgroup (median OS: 36.4 vs. 14.6 months, *p* < 0.001). Overall, the BTBC < 0% group also had significantly better OS than the BTBC ≥ 0% group (median OS: 36.5 vs. 8.5 months, *p* < 0.001) (Figure 3b).

**FIGURE 4:**
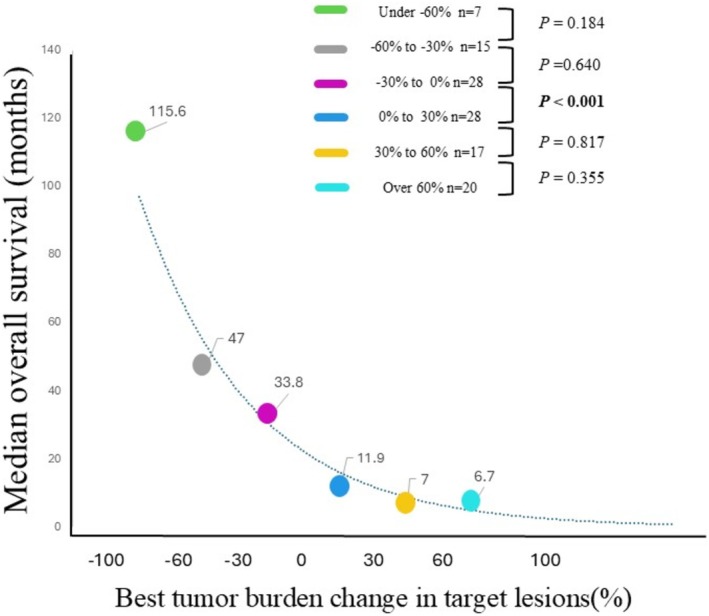
Comparison of median overall survival (OS) for each 30% interval of best tumor burden change (BTBC) in target lesions. The median OS for every 30% of BTBC was 115.6 months in the under −60% group (*n* = 7), 47.0 months in the −60% to −30% group (*n* = 15), 33.8 months in the −30% to 0% group (*n* = 28), 11.9 months in the 0% to 30% group (*n* = 28), 7.0 months in the 30%–60% group (*n* = 17), and 6.7 months in the over 60% group (*n* = 20). The only significant difference in OS was between the −30% and 0% group and the 0%–30% group (*p* < 0.001).

Additionally, the median OS stratified by every 30% BTBC intervals was as follows: 115.6 months for the under −60% group (*n* = 7), 47.0 months for the −60% to −30% group (*n* = 15), 33.8 months for the −30% to 0% group (*n* = 28), 11.9 months for the 0%–30% group (*n* = 28), 7.0 months for the 30%–60% group (*n* = 17), and 6.7 months for the over 60% group (*n* = 20) (Figure 4). A statistically significant difference in OS was only observed between the −30% to 0% group and the 0% to 30% group (*p* < 0.001).

In the BTBC < 0% group, only two patients (4%) had a tumor burden ≥ 0% in target lesions at the time of the first efficacy assessment, whereas 48 (96%) had a tumor burden < 0% from the initial assessment.

### 3.4 New Metastasis Lesions

**FIGURE 5:**
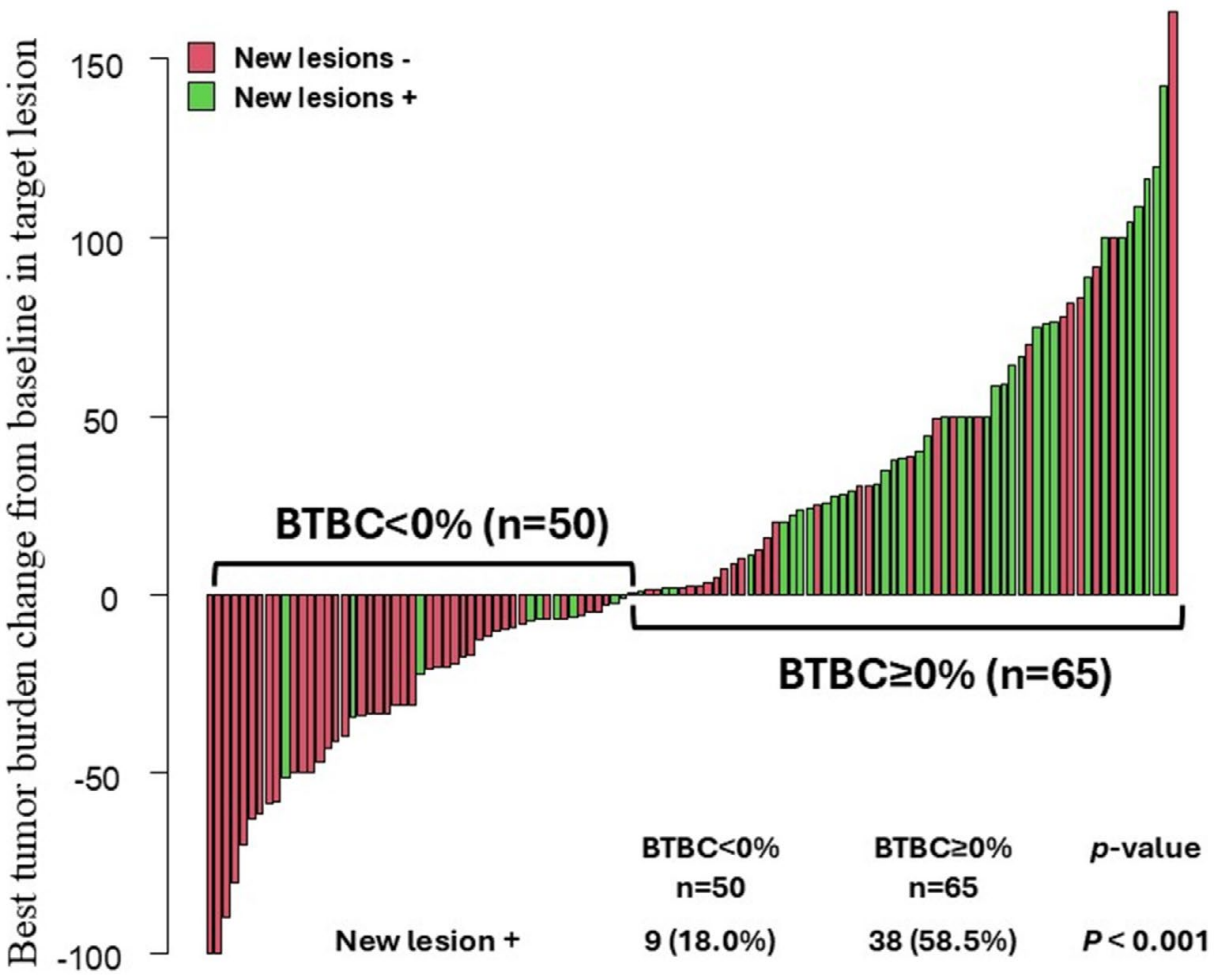
Waterfall plot of best tumor burden change (BTBC) in target lesions and new lesions in patients with malignant melanoma treated with anti‐PD‐1 monotherapy. The incidence of new lesions was significantly higher in the BTBC ≥ 0% group than in the BTBC < 0% group (58.5% vs. 18.0%, *p* < 0.001).

**FIGURE 6:**
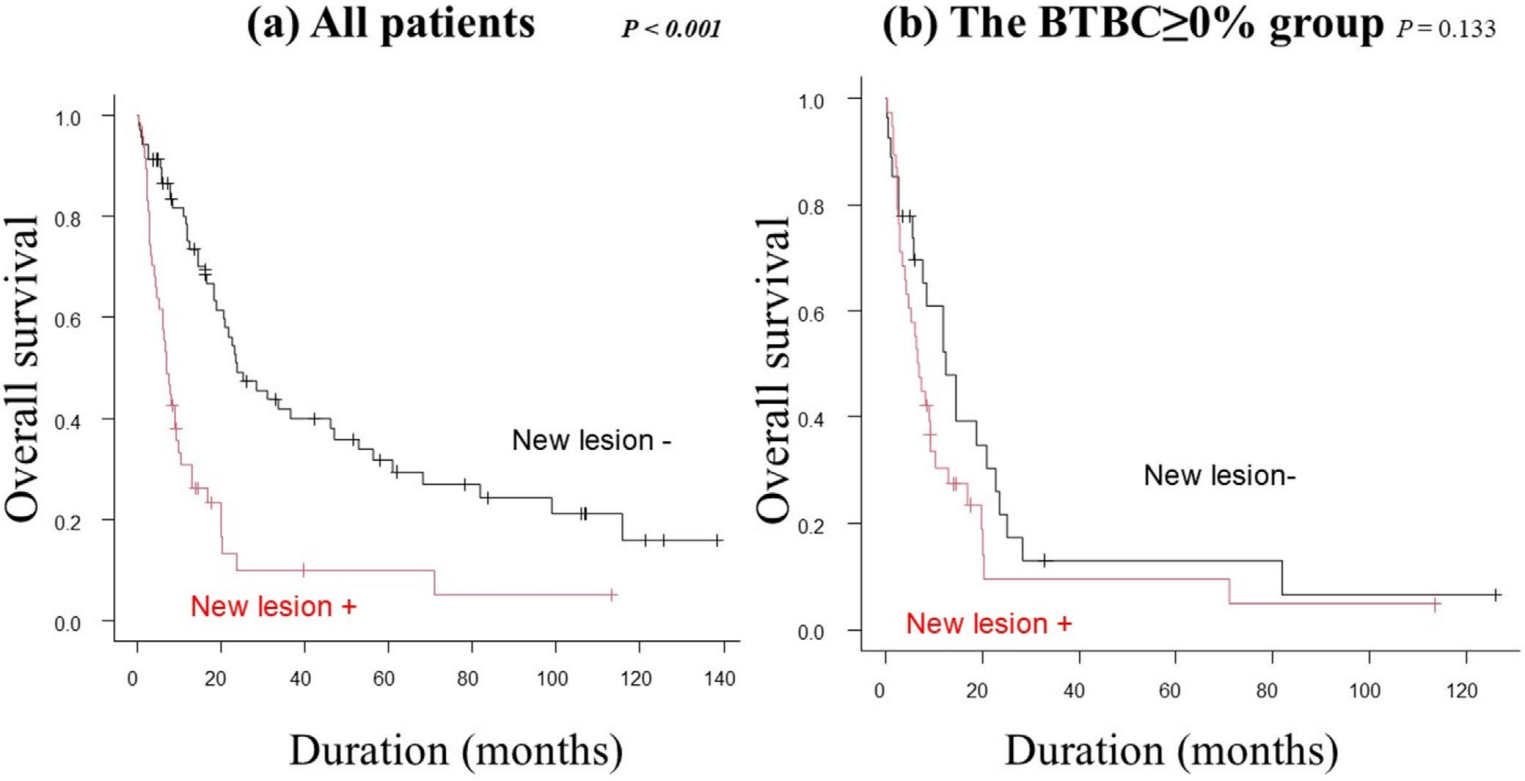
Kaplan–Meier analysis of overall survival (OS) according to the presence or absence of new lesions. (a) In all patients, those with new lesions had significantly worse OS than those without (median OS: 7.0 months vs. 23.8 months, *p* < 0.001). (b) In the BTBC ≥ 0% group, OS was not significantly different between patients with and without new lesions (median OS: 6.9 months vs. 12.6 months, *p* = 0.133).

The relationship between BTBC and the appearance of new metastatic lesions is shown in Figure 5. The incidence of new lesions was significantly higher in the BTBC ≥ 0% group than in the BTBC < 0% group (58.5% vs. 18.0%, *p* < 0.001). Among all patients, those with new lesions had a significantly worse OS than those without new lesions (median OS: 7.0 vs. 23.8 months, *p* < 0.001) (Figure 6a). However, within the BTBC ≥ 0% group, no significant difference in OS was observed between patients with and without new lesions (median OS: 6.9 vs. 12.6 months, *p* = 0.133) (Figure 6b).

### 3.5 Toxicity

**FIGURE 7:**
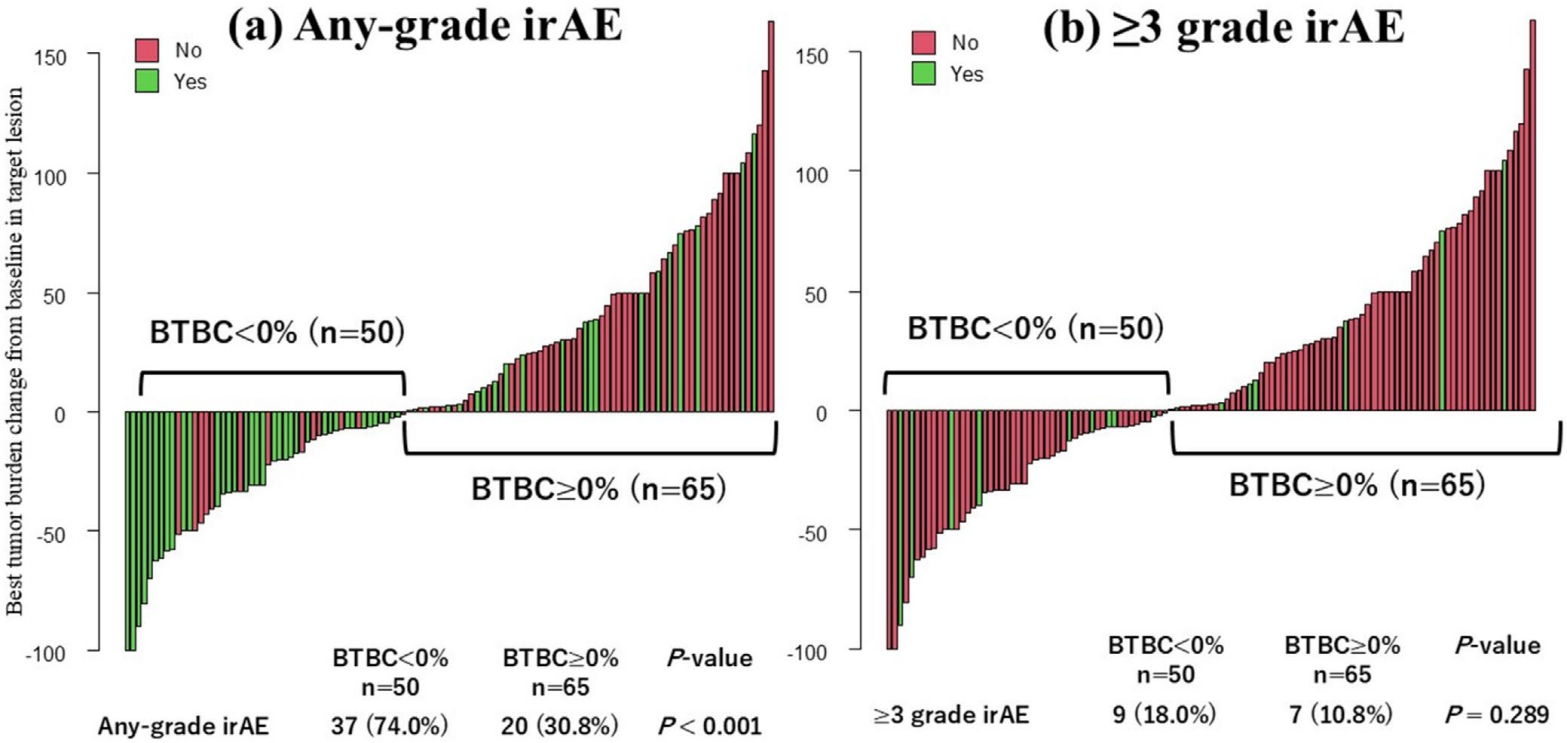
Waterfall plot of best tumor burden change (BTBC) in target lesions and the occurrence of immune‐related adverse events (irAEs) in patients with melanoma treated with anti‐PD‐1 monotherapy. (a) Waterfall plot for BTBC and any‐grade irAE. The incidence of any‐grade irAEs was significantly higher in the BTBC < 0% group than in the BTBC ≥ 0% group (74% vs. 30.8%, *p* < 0.001). (b) Waterfall plot for BTBC and ≥ 3 grade irAE. The incidence of ≥ 3 grade irAEs tended to be higher in the BTBC < 0 group than in the BTBC ≥ 0 group, although this difference was not statistically significant (18.0% vs. 10.8%, *p* = 0.289).

**FIGURE 8:**
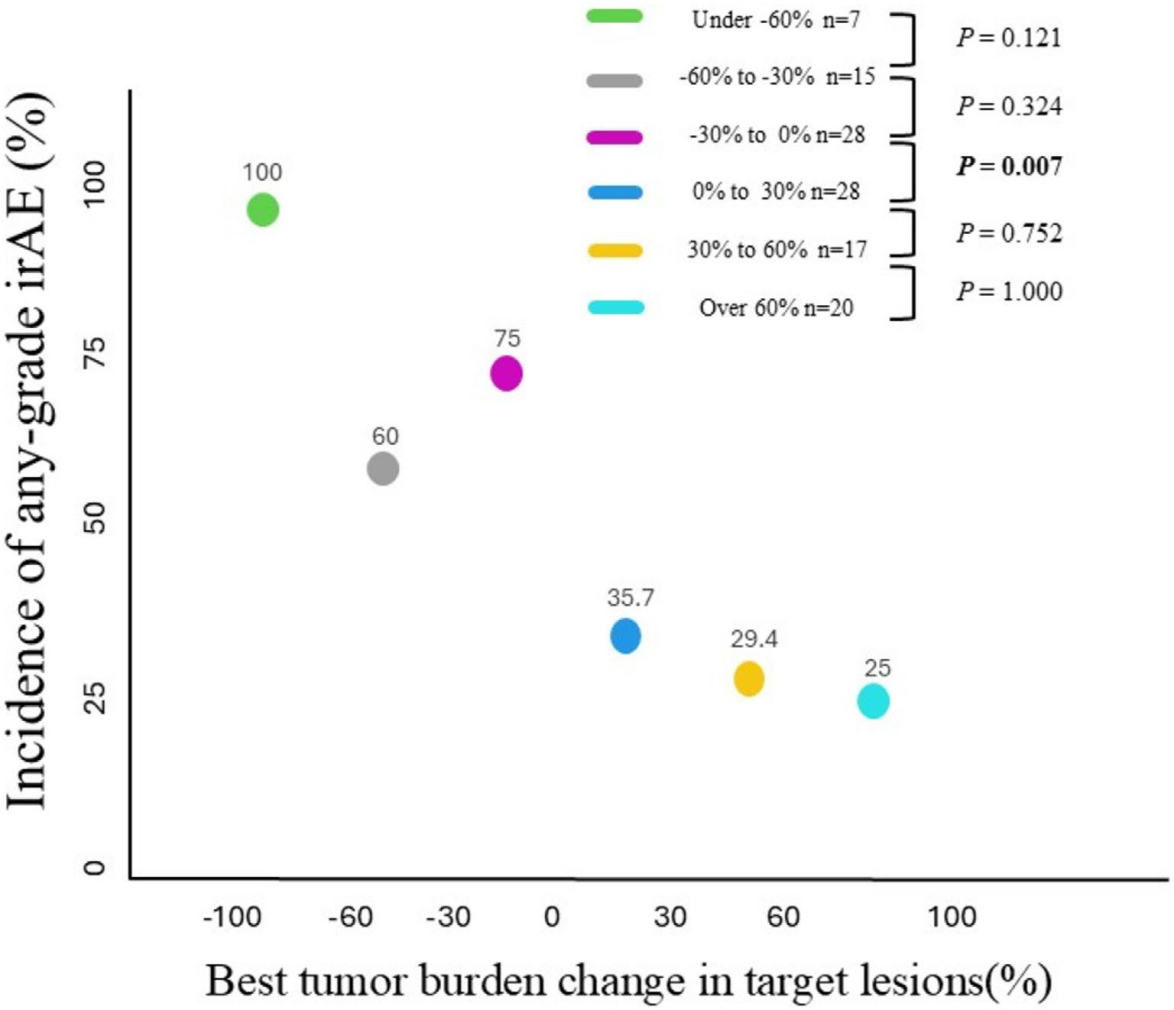
Comparison of the incidence of any‐grade immune‐related adverse events (irAEs) for each 30% interval of best tumor burden change (BTBC) in target lesions. The incidence of any‐grade irAEs for every 30% of BTBC was 100% in the under −60% group, 60% in the −60% to −30% group, 75% in the −30% to 0% group, 35.7% in the 0% to 30% group, 29.4% in the 30% to 60% group, and 25% in the over 60% group. A statistically significant difference was only found between the −30% to 0% group and the 0% to 30% group (*p* = 0.007).

The relationship between BTBC and irAEs is shown in Figure 7. The incidence of any‐grade irAEs was significantly higher in the BTBC < 0% group than in the BTBC ≥ 0% group (74% vs. 30.8%, *p* < 0.001) (Figure 7a). Although the incidence of ≥ 3 grade irAEs tended to be higher in the BTBC < 0% group than in the BTBC ≥ 0% group, the difference was not significant (18.0% vs. 10.8%, *p* = 0.289) (Figure 7b). Additionally, the incidence of any‐grade irAEs across each 30% BTBC interval was 100% in the under −60% group, 60% in the −60% to −30% group, 75% in the −30% to 0% group, 35.7% in the 0%–30% group, 29.4% in the 30%–60% group, and 25% in the over 60% group (Figure 8). A significant difference in the incidence of any‐grade irAEs was observed only between the −30% to 0% group and the 0%–30% group (*p* = 0.007).

To investigate the potential confounding effect of treatment duration, a logistic regression analysis was performed (Table 2). Univariate analysis showed that both BTBC < 0% (*p* < 0.001) and treatment duration (*p* = 0.008) were significantly associated with the occurrence of any‐grade irAEs. However, in the multivariate analysis, BTBC < 0% remained significantly associated with irAEs (*p* < 0.001), whereas treatment duration did not (*p* = 0.178).

**TABLE 2:** Univariate and multivariate analysis of potential prognostic factors for the incidence of any‐grade irAEs.

	Univariate analysis			Multivariate analysis		
	Odds ratio	95% CI	*p* ^a^	Odds ratio	95% CI	*p* ^a^
BTBC < 0%	Reference			Reference		
BTBC ≥ 0%	0.156	0.068–0.355	**< 0.001**	0.218	0.087–0.544	**0.001**
Treatment duration	1.000	1.000–1.000	**0.008**	1.000	1.000–1.000	0.178

Abbreviations: BTBC, best tumor burden change; CI, confidence interval; irAEs, immune‐related adverse events.

^a^ Bold values indicate statistical significance (*p* < 0.05).

## 4 Discussion

This study evaluated the association between BTBC and both prognosis and toxicity in patients with MM who received PD‐1 monotherapy as first‐line treatment. The key findings are as follows: First, prognosis improved in proportion to the reduction in tumor burden from baseline. Second, BTBC < 0% served as an indicator of a favorable long‐term prognosis. Third, the appearance of new lesions represented a poor prognostic factor, and BTBC ≥ 0% indicated poor prognosis regardless of new lesion development. Finally, the greater tumor shrinkage correlated with a higher incidence of adverse events.

Several reports have examined the relationship between BTBC and OS. A previous phase I clinical trial by Jain et al. [16] examined 468 patients with malignant tumors, including MM, who received systemic therapy. That study reported a nearly linear association between BTBC reduction and prolonged OS. These findings suggest that strict categorization into PR, SD, and progressive disease based on BTBC cutoffs (−30% and 20%) may be unnecessary. The authors proposed that using tumor response rates as continuous variables (e.g., waterfall plots) may provide more clinically relevant information than relying solely on best overall response classifications. Additionally, a sub‐analysis of the Checkmate067 study [17], a prospective trial evaluating the effects of ICIs in treatment‐naïve patients with MM, examined the association between tumor burden change and OS in those receiving nivolumab monotherapy. That sub‐analysis revealed minimal differences in Kaplan–Meier survival curves between patients with BTBC between −30% and −50% and those with SD.

Consistent with these findings, this study also showed no significant difference in OS between the PR and SD groups. These results, along with previous evidence, indicate that the traditional BTBC threshold of −30% does not hold practical relevance in clinical settings. Conversely, a statistically significant difference in OS was observed between the BTBC < 0% and BTBC ≥ 0% groups. Patients with BTBC < 0% demonstrated a linear inverse relationship between BTBC and OS, whereas among those with BTBC ≥ 0, prognosis remained poor regardless of the degree of BTBC. These results suggest that a BTBC of approximately 0% may function as a practical and intuitive cutoff value for predicting clinical outcomes (Figure 4). A BTBC of 0% represents the threshold between increasing and decreasing tumor burden and may serve as a clinically meaningful indicator of treatment efficacy.

One possible explanation for the poor prognosis among patients with a BTBC of ≥ 0% is the appearance of new lesions. Shibutani et al. [18] reported the prognostic significance of new lesions during the assessment of first‐line systemic therapy for metastatic colorectal cancer. In that study, among patients who experienced tumor progression, those who developed new lesions had significantly shorter OS than those who did not (*p* = 0.0068). In the present study, over half of the patients in the BTBC ≥ 0% group (58.5%) developed new lesions, and within the overall cohort, patients with new lesions exhibited significantly worse prognosis compared to those without (Figure 3b). However, although the appearance of new lesions in the BTBC ≥ 0% group tended to correlate with worse outcomes, it did not emerge as a statistically significant prognostic factor (Figure 3a). This lack of significance may reflect the small sample size and the aggressive nature of MM. The BTBC ≥ 0% group likely represents a more aggressive, less immunogenic tumor population intrinsically resistant to PD‐1 monotherapy. In this rapidly progressing disease state, the primary driver of poor prognosis appears to be the increasing tumor burden (BTBC ≥ 0%) itself, with new lesions arising as a consequence of this underlying biology. Given the tight association between the growth of existing lesions and the emergence of new ones in this group, isolating the independent prognostic impact of new lesions alone may have been statistically challenging. Therefore, BTBC ≥ 0% can be regarded as a key indicator of tumor aggressiveness and immunotherapy resistance, regardless of new lesion appearance. Notably, in all subgroups within the BTBC ≥ 0% group—specifically, the 0% ≤ BTBC < 30% group, the 30% ≤ BTBC < 60% group, and the BTBC ≥ 60% group—the OS remained below 1 year. Such uniformly poor outcomes may have diminished the discriminatory value of the 20% BTBC cutoff and the prognostic impact of new lesions.

Furthermore, 96% of patients in the BTBC < 0% group exhibited tumor shrinkage at the time of the initial efficacy evaluation. The reported frequency of pseudo‐progression in advanced MM treated with ICIs is 6.4%, a figure that aligns well with the findings in this study [19]. These data suggest that pseudo‐progression rarely occurs during PD‐1 monotherapy for advanced MM and that early prediction of prognosis is feasible using the initial efficacy evaluation. This study underscores the clinical value of stratifying patients based on BTBC values rather than relying solely on the best overall response. Nonetheless, these findings serve only as prognostic indicators. Prospective studies stratifying patients by BTBC cutoff values are required to determine whether PD‐1 monotherapy should be continued or discontinued based on tumor burden dynamics.

Regarding toxicity, this study observed a trend in which the incidence of any‐grade irAEs increased as BTBC decreased (Figures 7 and 8). Specifically, the incidence of any‐grade irAEs was significantly higher in the BTBC < 0% group compared to the BTBC ≥ 0% group (*p* < 0.001). Although the difference in the incidence of grade ≥ 3 irAEs did not reach statistical significance, this observation remains clinically relevant, as even low‐grade irAEs may serve as an indicator of an active and effective immune response. Several previous studies have explored the relationship between tumor response and irAEs in MM treated with PD‐1 monotherapy. Topalian et al. [20] conducted a prospective study evaluating the efficacy and side effects of nivolumab monotherapy in patients with MM, renal cell carcinoma, and non‐small cell lung cancer. That study included 270 patients, of whom 107 had MM. Among the MM cohort, patients who experienced any‐grade irAEs (*n* = 90) and ≥ 3 grade irAEs (*n* = 25) demonstrated significantly better ORRs than those who did not experience irAEs (*n* = 17) (*p* < 0.01 for both comparisons). Otsuka et al. [21] analyzed 27 patients with advanced MM who received nivolumab monotherapy and reported that patients who developed any‐grade irAEs tended to exhibit a better ORR (44% vs. 9%, *p* = 0.090) and disease control rate (81% vs. 18%, *p* = 0.002) compared to those who did not develop irAEs. Moreover, Nakamura et al. [22] studied 35 patients with MM treated with nivolumab and found that those who developed vitiligo had a significantly higher ORR than those who did not (44.4% vs. 7.7%, *p* = 0.027). Our results align with these findings and suggest that the occurrence of any‐grade irAEs, not only severe ones, may reflect the same underlying immune activation responsible for tumor shrinkage. To address the potential confounding effect of treatment duration, a multivariate analysis was performed. Initial univariate analyses demonstrated that both BTBC < 0% (*p* < 0.001) and treatment duration (*p* = 0.008) were significantly associated with irAEs. However, when both factors were included in a multivariate model, only BTBC < 0% retained a significant association (*p* < 0.001), whereas treatment duration did not (*p* = 0.178). This finding indicates that the apparent relationship between treatment duration and irAEs is likely a confounding effect driven by tumor shrinkage. Our data support the conclusion that irAEs are not merely a byproduct of prolonged therapy but may serve as a direct biomarker of an effective immune response leading to tumor regression. Therefore, the development of any‐grade irAEs, even minor ones, appears to be a valuable clinical indicator for monitoring the efficacy of PD‐1 monotherapy.

This study compared BTBC in target lesions with prognosis and the incidence of toxicity in patients with stage IV MM who received PD‐1 monotherapy. The findings suggested that, in clinical practice, BTBC may serve as a more accurate prognostic marker than best overall response. Additionally, a BTBC cutoff value of approximately 0 appeared to have prognostic utility. Because the BTBC < 0% group showed continued benefit from PD‐1 monotherapy and a higher incidence of irAEs, close monitoring of irAEs in these patients remains essential. Although these findings may support future clinical decision‐making, several limitations should be considered. First, this retrospective cohort design introduced the possibility of selection bias and may have led to underreporting of minor irAEs. Second, the retrospective nature of the study precluded the ability to draw definitive conclusions regarding whether to continue or change PD‐1 monotherapy based on BTBC. Thirdly, the single‐center setting and small sample size potentially limited the statistical power of the analysis. Finally, the selection of target lesions may have varied among evaluating physicians, introducing inter‐observer variability. To address these limitations, future prospective multicenter studies with larger cohorts are needed to validate these findings.

In conclusion, BTBC may serve as a more reliable indicator of treatment efficacy than best overall response in clinical practice for patients with stage IV MM receiving PD‐1 monotherapy. Future prospective studies are warranted to establish BTBC‐based criteria for guiding decisions on the continuation or discontinuation of PD‐1 therapy.

## Ethics Statement

Approval of the research protocol by an Institutional Review Board: Approved by the Ethics Committee of Shizuoka Cancer Center on June 23, 2025 (approval number: J2025‐73).

## Conflicts of Interest

The authors declare no conflicts of interest.

## Supporting information


**Figure S1:** jde17938‐sup‐0001‐FigureS1.docx.

## Data Availability

The data that support the findings of this study are available from the corresponding author upon reasonable request.
