# Supplementary material for: Prognosis and Toxicity Stratified by Best Tumor Burden Change in Japanese Patients With Advanced Melanoma Treated With First‐Line Programmed Cell Death Protein 1 Monotherapy
Source: J Dermatol. 2025 Sep 5;52(11):1656–65. doi: 10.1111/1346-8138.17938 (PMC12592579; doi:10.1111/1346-8138.17938)

**Supplementary materials**

**Supplementary Figure 1.** **Receiver operating characteristic curve of the optimal baseline tumor burden change (BTBC) cutoff value for predicting overall survival in patients with partial response and stable disease.**

The BTBC cutoff value with the lowest *P*-value for the difference in prognosis between the two groups was −2.8%, with a sensitivity of 0.326, specificity of 0.993, and area under the curve (AUC) of 0.649. The sensitivity and specificity of a BTBC cutoff value of 0% were 0.375 and 0.923, respectively.**
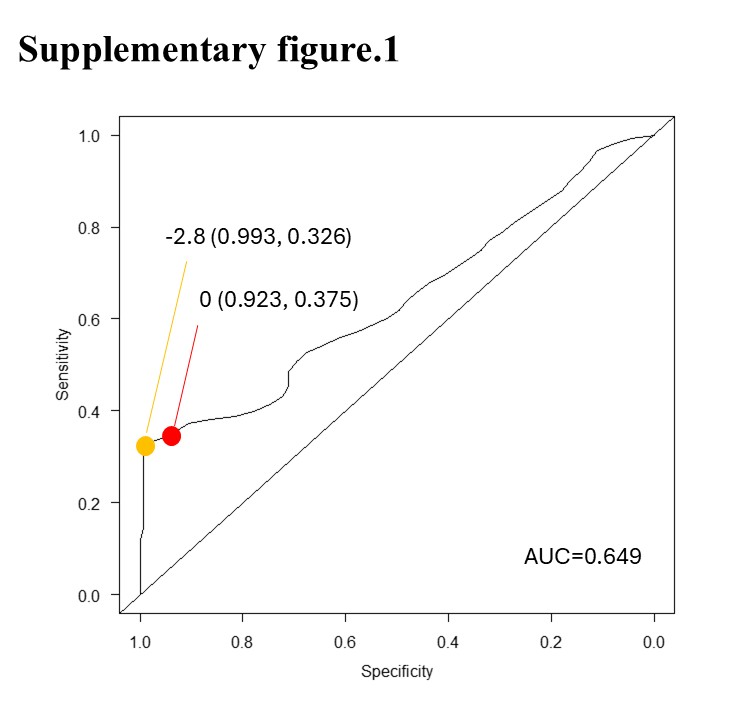
**

**Supplementary Figure 2.** **Receiver operating characteristic curve of the optimal baseline tumor burden change (BTBC) cutoff value for predicting overall survival in all patients.**

The BTBC cutoff value with the lowest *P*-value for the difference in prognosis between the two groups was −2.9%, with a sensitivity of 0.776, specificity of 0.766, and area under the curve (AUC) of 0.803. The sensitivity and specificity of a BTBC cutoff value of 0% were 0.712 and 0.690, respectively.
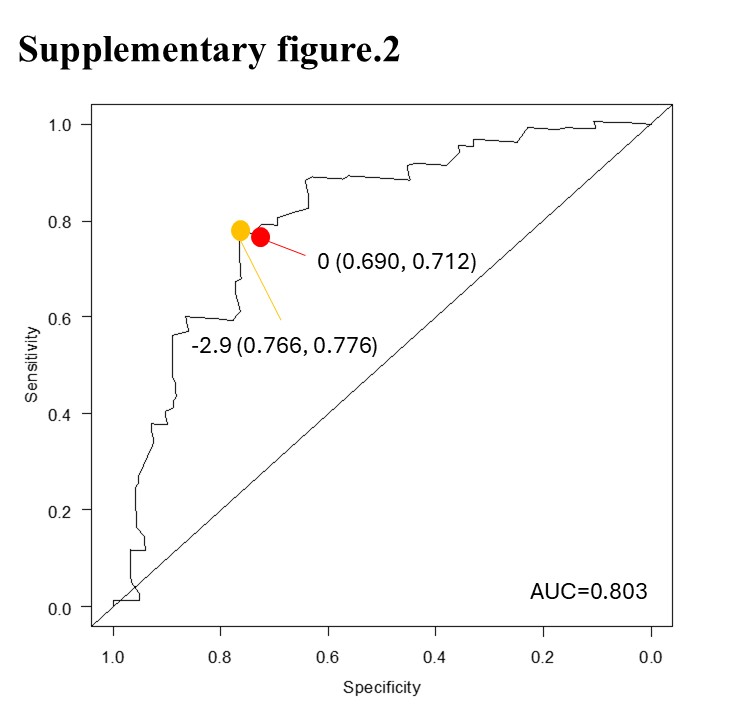

Supplement: Supplementary file 1 — Figure S1: jde17938‐sup‐0001‐FigureS1.docx. [file JDE-52-1656-s001.docx]
